# Typhus Group Rickettsiosis, Texas, USA, 2003–2013

**DOI:** 10.3201/eid2304.160958

**Published:** 2017-04

**Authors:** Kristy O. Murray, Nicole Evert, Bonny Mayes, Eric Fonken, Timothy Erickson, Melissa N. Garcia, Tom Sidwa

**Affiliations:** Baylor College of Medicine and Texas Children’s Hospital, Houston, Texas, USA (K.O. Murray, T. Erickson, M.N. Garcia);; Texas Department of State Health Services, Austin, Texas, USA (N. Evert, B. Mayes, E. Fonken, T. Sidwa)

**Keywords:** Typhus group, rickettsiosis, murine typhus, vector-borne infections, Texas, United States, fleas, bacteria, rickettsia, zoonoses

## Abstract

We characterized the epidemiology of typhus group rickettsiosis in Texas, USA. During 2003–2013, a total of 1,762 cases were reported to the state health department. The number of diagnosed cases and geographic expansion increased over time. Physician awareness is critical to diagnose and effectively treat rickettsial infections.

Typhus group rickettsiosis (TGR) is a fleaborne disease. In Texas, USA, most infections are attributed to *Rickettsia typhi*, the causative agent of murine typhus (*1*). Rare cases of another TGR, *R. prowazekii*, have been reported in south Texas (*2*).

The established reservoirs of murine typhus are *Rattus* spp. rodents; however, opossums are thought to be an important reservoir in peridomestic settings, with the cat flea, *Ctenocephalides felis*, as the vector (*3**–**5*). Clinical disease in humans often is characterized by the classical triad of fever, headache, and rash, although 1 study found that rash was present in only 54% of cases and only 12.5% had the classical triad (*6*). Infections can be severe and potentially fatal if not treated appropriately (*7*).

In the United States, Texas reports the most TGR cases annually, and TGR is considered endemic to the southernmost part of the state (*8*). Since the mid-2000s, public health authorities have observed an increase in the number of reported cases and geographic expansion into areas of the state to which TGR is not considered endemic. Our objective with this study was to characterize the epidemiology of TGR in Texas and identify high-risk geographic and demographic populations.

## The Study

The state of Texas mandates reporting of rickettsial diseases to the Texas Department of State Health Services (TxDSHS). TxDSHS maintains demographic, clinical, and environmental data on each case in a database for surveillance purposes. Confirmed cases of TGR were defined as clinically compatible illness with 1 of the following: 1) >4-fold rise in antibody titer by immunofluorescent antibody, complement fixation, latex agglutination, microagglutination, or indirect hemagglutination antibody between acute and convalescent specimens; 2) a positive PCR result; 3) bacterial isolation from a clinical specimen; 4) positive immunofluorescence from tissue; or 5) a single IgM or IgG titer of >1,024 in the TGR-endemic area of south Texas or Travis County, beginning in 2007 and 2012, respectively. Probable cases were defined as clinically compatible illness and a single serologic titer of >128 by immunofluorescent antibody, latex agglutination, microagglutination or indirect hemagglutination antibody or a single titer of >16 by complement fixation.

We analyzed surveillance data on all confirmed and probable cases reported to TxDSHS during 2003–2013. Census data from the 2010 national census (*9*) were used to derive attack rates for sex, age, and race/ethnicity, as well as cumulative incidence by county. Statistical estimates were calculated by using Epi Info version 7.2 (Centers for Disease Control and Prevention [CDC], Atlanta, GA, USA). Incidence by county was graphed using MapInfo version 10.2.5 (Pitney Bowes Software, Troy, NY, USA).

**Figure 1:**
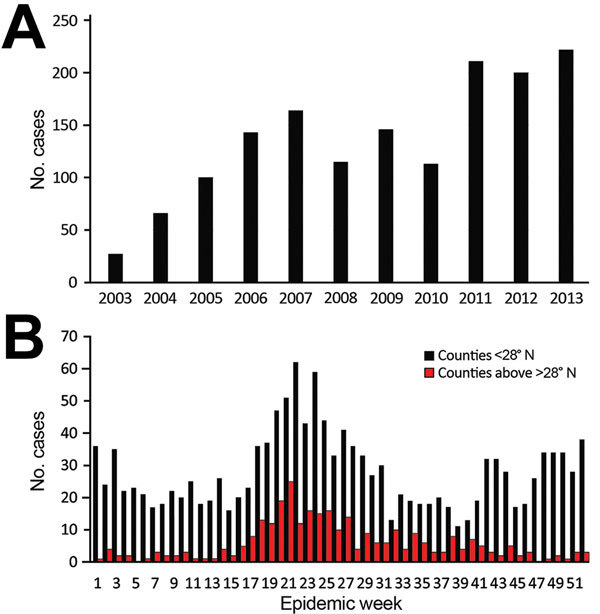
Typhus group rickettsiosis, Texas, 2003–2013. A) Number of reported cases by year. B) Illness onset by epidemic week during the study period, by location; <28°N represents south Texas.

During 2003–2013, a total of 1,762 TGR cases (770 confirmed and 992 probable) were reported. We observed evidence of increased numbers of cases over time and expanded geographic locations. We know of no specific reason that would have prompted physicians to increase diagnosing or reporting cases. During the study period, case numbers reported ranged from 27 in 2003 to 222 in 2013 (Figure 1). An average of 102 cases were reported yearly during 2003–2007, which is less than half (209) of the average number reported during 2008–2013. As expected, illness onset peaked in June and July; however, in south Texas (<28°N), 2 peaks occurred: the first in summer (June and July) and the other in winter (December and January). The reason for this bimodal distribution of cases in south Texas is unknown and requires further investigation.

**Figure 2:**
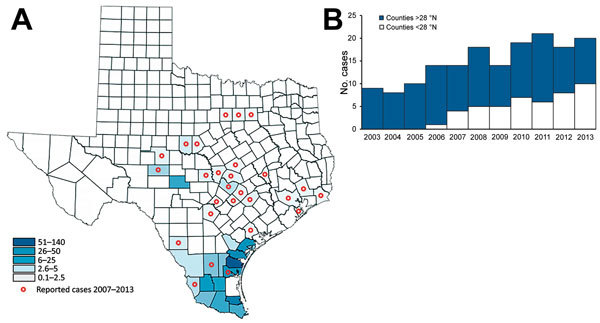
Geographic distribution of typhus group rickettsiosis, Texas, 2003–2013. A) County-level cumulative incidence per 100,000 population, 2003–2013, and spread into new geographic areas beginning in 2007. B) Incidence stratified by geographic location; <28°N represents south Texas.

TGR cases expanded geographically during the study period. In 2003, cases were reported from 9 counties in south Texas. By 2013, cases had been reported from 41 counties (Figure 2). Cumulative incidence was highest in south Texas; an average of 59.5 cases per 100,000 population were reported during the study period (Figure 2). Nueces County in south Texas had the highest cumulative incidence (139.9 cases/100,000 population). One county (Kenedy) in south Texas reported no cases; this is most likely due to this county’s low population count (416 persons) (*10*).

The attack rate was slightly higher for female than for male residents (7.3 vs. 6.7/100,000 population) (Table 1). Median age of case-patients was 33 years, and the highest attack rate was for 5–19-year-olds (10.4 cases/100,000 population). These findings contrast with a 1980s study of 345 murine typhus case-patients in south Texas for whom median age was 48 years, and only 5 (1.4%) case-patients were <11 years of age (*6*).

**Table 1:** Demographic characteristics and attack rates for typhus group rickettsiosis, Texas, 2003–2013

Characteristic	Cases, no. (%), n = 1,762	Census population*	Attack rate†
Sex			
F	921 (52)	12,673,281	7.3
M	841 (48)	12,472,280	6.7
Race/ethnicity			
White, non-Hispanic	429 (24)	11,397,345	3.8
White, Hispanic	1,133 (64)	9,460,921	12.0
Black	23 (1)	2,886,825	0.8
Unknown or other	177 (10)	1,400,470	12.6
Age group, y			
<5	40 (2)	1,928,473	2.1
5–19	591 (34)	5,693,241	10.4
20–39	398 (23)	7,194,139	5.5
40–64	575 (33)	7,727,822	7.4
>65	158 (9)	2,601,886	6.1

*Based on 2010 Population Census for Texas. †Per 100,000 population.

Fifty-four percent of case-patients reported fleas in the home, and 34.0% reported a flea bite before illness onset (Table 2). The most common animals in the home environment were dogs (67.0%), cats (46.2%), wildlife (42.4%), and rodents (28.9%); 57.7% of homes had >1 animal.

**Table 2:** Clinical and environmental characteristics of reported TGR cases, Texas, 2003–2013*

Characteristic	Total, no. (%)	Confirmed case,† no. (%)	Probable case,‡ no. (%)
Clinical features			
Hospitalized	1,047/1,758 (59.6)	473/767 (61.7)	574/991 (57.9)
Fever	1,747/1,752 (99.7)	759/764 (99.3)	988/988 (100)
Headache	1,353/1,753 (77.2)	569/765 (74.4)	784/988 (79.4)
Chills	1,228/1,753 (70.1)	511/765 (66.8)	717/988 (72.6)
Malaise	1,123/1,753 (64.1)	471/765 (61.6)	652/988 (66.0)
Anorexia	925/1,753 (52.8)	399/765 (52.2)	526/988 (53.2)
Nausea/vomiting	901/1,753 (51.4)	381/765 (49.8)	520/988 (52.6)
Myalgia	890/1,753 (50.8)	388/765 (50.7)	502/988 (50.8)
Rash	722/1,700 (42.5)	325/747 (43.5)	397/953 (41.7)
Diarrhea	394/1,753 (22.5)	184/765 (24.1)	210/988 (21.3)
Photophobia	381/1,752 (21.7)	160/765 (20.9)	221/987 (22.4)
Retroorbital pain	266/1,753 (15.2)	121/765 (15.8)	145/988 (14.7)
Classical triad (fever, headache, rash)	572/1,697 (33.7)	240/745 (32.2)	332/952 (34.9)
Abnormal laboratory findings			
Elevated liver function	478/1,751 (27.3)	192/765 (25.1)	286/986 (29.0)
Thrombocytopenia	290/1,752 (16.6)	123/765 (16.1)	167/987 (16.9)
Environmental findings			
Fleas present in home	707/1,310 (54.0)	313/580 (54.0)	394/730 (54.0)
History of flea bite	418/1,231 (34.0)	187/538 (34.8)	231/693 (33.3)
Rodents present	379/1,312 (28.9)	192/584 (32.9)	187/728 (25.7)
Wildlife present	587/1,383 (42.4)	273/617 (44.2)	314/764 (41.1)
Dogs present	973/1,452 (67.0)	411/640 (64.2)	562/809 (69.5)
Cats present	659/1,427 (46.2)	298/633 (47.1)	361/792 (45.6)
>1 species present	844/1,463 (57.7)	371/644 (57.6)	472/816 (57.8)

*Denominators are the number of case-patients for whom the information was available. CF, complement fixation; IFA, immunofluorescent antibody; IHA, indirect hemagglutination antibody; LA, latex agglutination; MA, microagglutination; TGR, typhus group rickettsiosis. †A confirmed case was defined as clinically compatible illness with 1) a >4-fold rise in antibody titer by IFA, CF, LA, MA, or IHA between acute and convalescent specimens; 2) a positive PCR result; 3) bacterial isolation from a clinical specimen; 4) positive immunofluorescence from tissue; or 5) a single IgM or IgG titer of >1,024 in a TGR-endemic region of south Texas (beginning in 2007) or Travis County (beginning in 2012). ‡A probable case was defined as clinically compatible illness and a single serologic titer of >128 by IFA, LA, MA, or IHA or a single titer >16 by CF.

A total of 1,047 (59.6%) case-patients were hospitalized (Table 2). The most commonly reported signs and symptoms were fever (99.7%), headache (77.2%), chills (70.1%), malaise (64.1%), anorexia (52.8%), nausea/vomiting (51.4%), and myalgias (50.8%). Rash was reported in 42.5% of cases; children were more likely than adults to have a rash (54.5% vs. 35.7%; odds ratio 2.2, p<0.001). One third (33.7%) of case-patients had the classical triad of fever, headache, and rash, which was higher than the previously reported 12.5% by Dumler et al. (*6*). Children were more likely than adults to have all 3 symptoms (41.1% vs. 29.3%; p<0.001). Four deaths were reported (0.2% case-fatality rate). The median age of persons who died was 51.5 years (range 36–55 years).

Identification and reporting of *Rickettsia*-positive persons is critical to identifying disease outbreaks. Although TGR is reportable in Texas, our study probably underrepresents the true number of cases because the illness was mild or not recognized as TGR, the clinical provider did not realize the disease is reportable, or the disease was misclassified (false negative) because specimens were collected too early after symptom onset (*11*).

Another potential limitation is the propensity of cross-reactions with other rickettsial pathogens. We would expect some degree of serologic cross-reaction between the 2 pathogens within the TGR group, *R. typhi* and *R. prowazekii*; hence, we collectively call these diagnoses TGR, even though we presume most cases were attributed to *R. typhi* infections. Serum reactive to typhus group antigen might cross-react, albeit infrequently and at a lower titer, to spotted fever group antigen (*12*). Finally, until 2015, TxDSHS included in the case definition for a confirmed case a single IgM titer >1,024 in TGR-endemic areas. This decision was based on CDC’s criteria for diagnosing spotted fever rickettsiosis (*13*). As mentioned by CDC, a single IgM result is not ideal for diagnosing acute infections due to reduced specificity. Further research is needed to understand the true incidence of TGR in Texas.

## Conclusions

We observed increased cases and geographic spread of TGR in Texas. Our results highlight the importance of educating the public about flea-bite prevention and raising physician awareness to identify cases, particularly in children. Further research is needed to better understand the transmission dynamics between rodents, fleas, and other potential new reservoir/vector systems and determine risk factors for infection.
